# High-dose dexamethasone injection disordered metabolism and multiple protein kinases expression in the mouse kidney

**DOI:** 10.1042/BSR20211847

**Published:** 2021-11-18

**Authors:** Zaikuan Zhang, Yingchun Ran, Lei Xu, Zheng Pan, Yajun Xie

**Affiliations:** 1 The M.O.E. Key Laboratory of Laboratory Medical Diagnostics, The College of Laboratory Medicine, Chongqing Medical University, Chongqing 400016, P.R. China; 2 Department of Emergency Children’s Hospital of Chongqing Medical University, National Clinical Research Center for Child Health and Disorders, Ministry of Education Key Laboratory of Child Development and Disorders, Chongqing Key Laboratory of Pediatrics, Chongqing 400014, P.R. China; 3 The College of Traditional Chinese Medicine, Chongqing Medical University, Chongqing 400016, P.R. China

**Keywords:** dexamethasone, Glycolysis, Kidney, PPAR, RNA-Seq

## Abstract

Glucocorticoids (GCs) have been widely used in clinical treatment as anti-inflammatory, anti-shock and immunosuppressive medicines. However, the effect of excessive GCs on immune response and metabolism of kidney remains unclear. Here, we profiled the gene expression of kidney from mice with high-dose dexamethasone (DEX) treatment. A total of 1193 differentially expressed genes (DEGs) were screened in DEX treatment group compared with the saline group, including 715 down- regulated and 478 up-regulated. Gene Ontology (GO) and Kyoto Encyclopedia of Genes and Genomes (KEGG) pathway analyses of these DEGs showed extracellular matrix (ECM)–receptor interaction, cell adhesion molecules signaling pathway were significantly enriched, and that the vast majority of DEGs were involved in monocarboxylic acid metabolism, leukocyte cell–cell adhesion and fatty acid metabolism. Gene set enrichment analysis (GSEA) revealed that DEGs were strongly associated with immune-response and cell adhesion gene sets, such as Fc γ R-mediated phagocytosis, leukocyte transendothelial migration, T-cell receptor signaling pathway, cell adhesion, ECM–receptor interaction and focal adhesion-associated pathways. KEGG pathway analysis of differentially expressed kinases (DEKs) showed T-cell receptor and forkhead box class O signaling pathway were enriched. Furthermore, we found multiple protein kinases expression were dysregulated greatly after dexamethasone treatment, including classical effector of GCs stimulation-serum and GC-regulated kinase. These protein kinases are involved in multiple signaling pathways in mice kidney, such as mitogen-activated protein kinase (MAPK) and phosphoinositide 3-kinase (PI3K)/Akt signaling pathway. We profiled the gene expression of the kidney from high-dose dexamethasone-treated mice and provided important information for further study the mechanism of side effects of GCs in clinical therapy.

## Introduction

Glucocorticoids (GCs), a key regulatory hormone in the human body and functioning in homeostasis and development [1], are widely used in clinical treatment as an anti-inflammatory, anti-shock and immunosuppressive agent [2,3]. Given its anti-inflammatory cytokine property, GCs have become the most frequently prescribed drugs in autoimmune, inflammatory and allergic diseases, such as rheumatology [4], septic shock [5], inflammatory bowel disease [6] and so on. Low doses of GCs are clinically recommended for the treatment of septic shock [7] and rheumatic diseases [8]. However, GCs are like a double-edged sword. Owing to a series of deleterious side effects that may occur after long-term or high-dose usage of GCs, such as osteoporosis, hyperglycemia, insulin resistance, disturbed fat deposition, hypertension and muscle atrophy [9–14], the amount and duration of GCs administration need to be carefully adjusted. Because of a low therapeutic index, GCs are prone to exert a lot of time- and dose-dependent side effects, the causes of which are not well understood [15]. Thus, a complete understanding of side effects and pathogenic mechanisms of long-term and high-dose treatment is essential to determine the appropriate use of these drugs.

The kidney plays an essential role in removing metabolic waste from the blood and maintaining electrolyte balance [16] and the amount of GCs is vital for maintaining kidney functions. Clinically, GCs are used to treat a variety of kidney diseases, including minimal change disease, idiopathic focal segmental glomerular sclerosis, Immunoglobulin A nephropathy, Membranous nephropathy, complement C3 glomerulopathy, pauci-immune rapidly progressive glomerulonephritis and lupus nephritis [17]. Meanwhile, the appropriate GCs have been reported to ameliorate acute kidney injury induced by renal ischemia–reperfusion injury [18] and reduce proteinuria by inhibiting the focal adhesion kinase (FAK)/receptor activator of nuclear factor-κB ligand (RANKL)/mitogen-activated protein kinase (MAPK) and FAK/RANKL/nuclear factor κβ (NF-κB) signaling pathways in rats with adriamycin-induced nephropathy [19]. Nevertheless, with the increasing popularity of GC in the treatment of various kidney diseases, more and more attention has been paid to the side effects of GC use. However, the mechanisms underlying deleterious side effects of excessive use of GCs remain unclear.

In the present study, we intraperitoneally injected mice with high-dose dexamethasone (DEX) and performed RNA sequencing (RNA-seq) to identify differentially expressed genes (DEGs) in mice kidneys. Gene Ontology (GO) and Kyoto Encyclopedia of Genes and Genomes (KEGG) enrichment were analyzed to understand functions of DEGs after high-dose GCs treatment, which further elucidating the mechanism of side effects for long-term and high-dose GC treatment and providing evidence for clinical practice.

## Materials and methods

### Mice and DEX treatment

All animal studies were reviewed and approved by the Institutional Animal Care and Use Committee (IACUC) of Chongqing Medical University (Reference Number: 2018020, date approved: 6 June 2018). All mice were maintained in a special pathogen-free facility. All animal experiments were conducted at the Animal Center of Chongqing Medical University (Chongqing, China) and all efforts were made to minimize animal suffering. Specific pathogen-free (SPF) grade C57BL/6 male mice were obtained from the Animal Center of Chongqing Medical University. C57BL/6 mice with body weight 22–24 g were randomly divided into the control group (*n*=6) and DEX treatment group (*n*=6). Mice of the high-dose treatment group were intraperitoneally injected with 10 μg/g body weight/day DEX for 1 week (TopHat: discovering splice junctions with RNA-Seq). Mice of the control group were intraperitoneally injected with an equal volume of physiological saline (0.9% NaCl) for 1 week.

### Sample collection

Mice were anesthetized with chloral hydrate (400 mg/kg) by intraperitoneal injection. Subsequently, kidneys were removed and weighed, and then the mice were killed by cervical dislocation. The kidney coefficient, which represented the relative kidney size, was the ratio between the average kidney weight and body weight. Partial kidney tissues were frozen in liquid nitrogen for transcriptome sequencing and stored at −80°C for ribonucleic acid (RNA) isolation. The residual kidney tissues were fixed with 4% paraformaldehyde at 4°C for 24 h.

### RNA isolation and real-time quantitative polymerase chain reaction

Total RNA of mice kidney was extracted with TRIzol (Invitrogen, Grand Island, NY) and RNA concentration was checked using the NanoDrop spectrophotometer (Thermo Scientific, Waltham, MA, U.S.A.). Ribosomal 28S and 18S RNA were used to assess the quality of RNA after agarose electrophoresis. Two microgram RNA was reverse-transcribed using the First-Strand cDNA Synthesis kit (Thermo Scientific, Waltham, MA, U.S.A.) according to the manual. Real-time quantitative polymerase chain reactions (qPCRs) were carried out using Ultra-SYBR Mixture (CWBIO, Beijing, China) on a CFX Connect™ sequence detector (Bio-Rad, California, U.S.A.). 18S was used as control. The primers for quantitative PCR are shown in Supplementary Table S1. Each experiment was repeated three times. All of the data are presented as mean ± s.e.m. The statistical significance of differences was analyzed by the Student’s *t* test, *P<0.05, **P<0.01, ***P<0.001.

### Library preparation for transcriptome sequencing

NEB Next® Ultra™ Directional RNA Library Prep Kit for Illumina® (NEB, U.S.A.) was used to construct an mRNA-based library according to the user guide. Index codes were added for tracking. In short, mRNA was isolated from total RNA by NEB Next poly(A) mRNA Magnetic Isolation Module (NEB #E7490) containing oligo-dT beads. The isolated mRNA was chemically fragmented using divalent cations in NEB 5× Next First Strand Synthesis Reaction Buffer. Random hexamer primers and M-MuLV Reverse Transcriptase (RNase H-) were used to synthesize first strand complementary DNA (cDNA). Second strand cDNA was synthesized using DNA polymerase I and RNase H. Terminals of double strand cDNA were repaired by exonuclease/polymerase. After 5′ ends of the deoxyribonucleic acid (DNA) fragments were phosphorylation modified and 3′ ends of the DNA fragments were modified with adenylation, the NEB Next Adaptors for Illumina platform were ligated to ends of the cDNA. cDNA fragments of 150–200 bp were selected and purification was performed using the AMPure XP system (Beckman Coulter, Beverly, U.S.A.). PCR was carried out to amplify cDNA fragments. PCR products were purified and the quality of DNA library was evaluated with the Agilent Bioanalyzer 2100 system.

### Transcriptome sequencing and expression analysis

The constructed DNA library was sequenced using the Illumina Hiseq 4000 sequencing platform (Illumina) following the manufacturer’s instructions for 150-bp paired-end reads (Novogene, Beijing, China). The reads which contained adapter sequences, low-quality bases and undetermined bases were removed to obtain clean reads. Reference genome and gene model annotation files were downloaded from the genome website (ftp://ftp.ensembl.org/pub/release-84/fasta/mus_musculus/). Reads were then aligned to *Mus musculus* genome (version mm10) using Bowtie v2.2.7 [20], rejecting reads that contain more than a single mismatch.

Since expression values approximated a log-normal distribution, fragments per kilobase of transcript per million mapped fragments (FPKM) of each gene was floored to 1, and log2-transformed for further analysis. DEGs were then identified using DEseq2 package (v1.22.1) [21] of R software (v3.6.3). *P*-values were adjusted using Benjamini and Hochberg’s approach [22] to control the false discovery rate. Genes with an adjusted *P*-value <0.05 and |logFC| ≥1 were identified as DEGs. Venn diagrams based on the gene list of different groups were drawn using VennDiagram package (v1.6.17) in R. Gene expression profile of DEGs was presented in the form of heatmap using Pheatmap R package (v1.0.12).

### GO, KEGG enrichment and gene set enrichment analysis

GO is a gene function classification system that provides a set of standard vocabulary to describe the attributes of genes and gene products in organisms [23]. GO enrichment analysis of DEGs was conducted using the GOseq R package (v1.44.0) [24]. *P*-values were adjusted by the method of Benjamin–Hochberg. GO terms which satisfied adjusted *P*-value <0.05 were thought to be significantly gene enriched. KEGG (https://www.genome.jp/kegg/) is a database resource for understanding high-level functions and utilities of the biological system from molecular level information, especially in the format of large-scale molecular datasets generated by genome sequencing and other high-throughput experimental technologies [25]. KEGG enrichment of DEGs was identified using KOBAS 3.0 software, and pathways were filtered by adjusted *P*-value/FDR < 0.05. Gene set enrichment analysis (GSEA) is utilized to analyze the differential expression of relevant sets of genes which perform similar biological functions [26]. Hallmark pathway datasets (h.all.v7.4.symbols.gmt) were used for GSEA in the present study. Gene sets which satisfied an adjusted *P*-value <0.05 and a normalized enrichment score > 1 were considered significantly enriched.

### Protein–protein interaction network analysis

STRING (https://string-db.org) depicts a physical and functional protein–protein interaction (PPI) networks based on existing knowledge and predictions through systematic co-expression analysis and text-mining of literature [27]. PPI analysis of DEGs was carried out on the basis of the STRING protein interaction database with a minimal confidence score of 0.4.

### Kidney histology

Mouse kidney tissues were fixed in 4% paraformaldehyde, embedded in paraffin and sectioned at 5 μm for analysis. Sections were dewaxed in xylene and rehydrated through gradient ethanol, and observed using Hematoxylin and Eosin (H&E) staining. The sections were photographed with a microscope (DM4B, Leica, Germany).

### Statistical analysis

Statistical analysis was conducted by GraphPad Prism 8.0.2, and data are expressed as mean ± s.e.m. Statistical significance was analyzed by Student’s *t* test and defined as a *P*-value <0.05.

## Results

### DEX treatment affected the expression of genes involved in metabolism, inflammation and immune response

**Figure 1:**
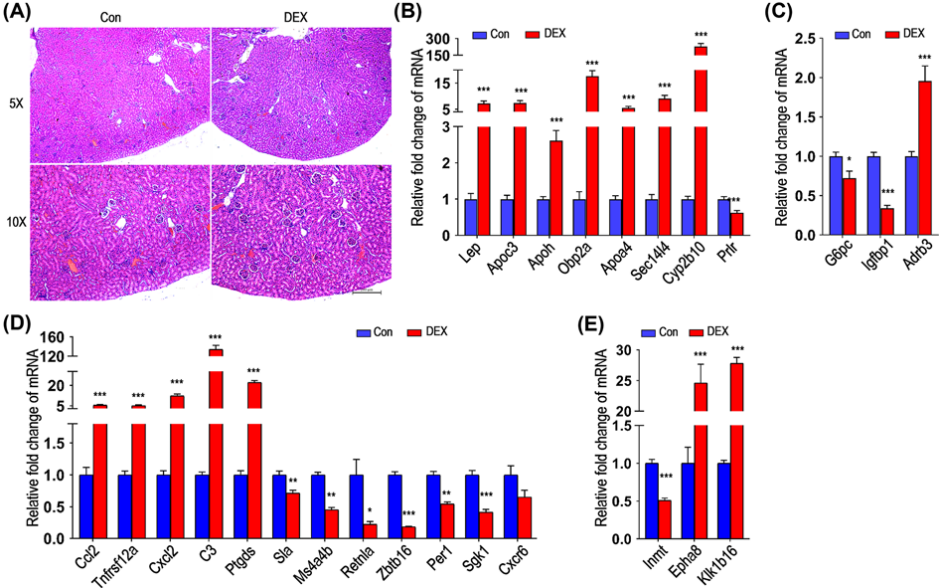
Validation analysis of metabolism, inflammation and immune-associated genes in kidneys of mice with DEX treatment (**A**) Kidney section of control (Con, left) and experiment group (DEX, right). These sections were stained with H&E, scale bar: 200 μm (*n*=6). Shown are representative images. (**B–E**) The transcriptional levels of genes related to lipid metabolism (B), glucose metabolism (C), immune (D) and other functions (E) were measured by quantitative PCR (blue: control, red: DEX; mean ± s.e.m; *n*=3, *P<0.05, **P<0.01, ***P<0.001, Student’s *t* test).

We first tested whether high doses of DEX affected body weight and kidneys in mice. The data (Supplementary Figure S1A–C) showed that there was no difference in body weight and kidney weight between the experiment and control groups. Studies in rodents, sheep, and non-human primates suggested that corticosteroids might harm the kidneys by significantly reducing the number of nephrons [28]. To investigate the effects of excessive GC treatment on the kidneys, we performed kidney histological studies to detect the potential alterations in kidney morphology. H&E staining of paraffin-embedded kidney tissues indicated no pathological changes in the experimental group compared with the control group (Figure 1A). Energy substrates (such as glucose, amino acids and fatty acids) could be released by GCs, thus ensuring that they are oxygenated by the mitochondria. However, long-term GCs overexposure alters expansion of trunk adipose tissue depots and impairs metabolism and insulin action, resulting in hyperglycemia and dyslipidemia [29]. Our data showed that lipid metabolism-associated genes, such as leptin (*Lep*), apolipoprotein c3 (*Apoc3*), apolipoprotein H (*Apoh*), odorant-binding protein 2a (*Obp2a*), apolipoprotein a4 (*Apoa4*), SEC14-like lipid binding 4 (*Sec14l4*) and cytochrome P450 family 2 subfamily B member 10 (*Cyp2b10*) were up-regulated, whereas prolactin receptor (*Prlr*), reducing the speed limit enzyme stearyl coenzyme A desaturation enzyme 1 in the biosynthesis of monounsaturated fatty acids, was down-regulated after DEX treatment (Figure 1B), indicating that long-term and high-dose GCs induced lipid metabolism disorders. Moreover, glucose metabolism-associated genes, glucose-6-phosphatase (*G6pc*) and insulin-like growth factor binding protein 1 (*Igfbp1*), were down-regulated, accompanied with adrenoceptor β 3 (*Adrb3*) whose gene polymorphism is related to type 2 diabetes and obesity up-regulated (Figure 1C). GCs have powerful anti-inflammatory functions and immunosuppressive effects. Our data showed that pro-inflammatory genes including C–C motif chemokine ligand 2 (*Ccl2*), tumor necrosis factor receptor superfamily member 12A (*Tnfrsf12a*), C–X–C motif chemokine ligand 2 (*Cxcl2*), complement C3 (*C3*) and prostaglandin D2 synthase (*Ptgds*), which indicated the activation of inflammatory response with DEX treatment, were up-regulated after long-term and high-dose GCs use. However, transcripts of T-cell signaling inhibition related genes Src-like adaptor (*Sla*), membrane-spanning 4-domain family subfamily A member 4b (*Ms4a4b*), T helper cell regulator (*Retnla*) and zinc finger and BTB domain containing 16 (*Zbtb16*), which affects the effector function of natural killer (NK) cells, were down-regulated, coupled with period circadian regulator 1 (*Per1*), serum-glucocorticoid regulated kinase 1 (*Sgk1*) and C–X–C motif chemokine receptor 6 (*Cxcr6*) down-regulated (Figure 1D). At the same time, the expression of indolethylamine N-methyltransferase (*Inmt*) decreased, whereas erythropoietin-rroducing hepatoma receptor A8 (*Epha8*) and kallikrein 1-related peptidase b16 (*Klk1b16*) increased significantly (Figure 1E). Taken together, these results indicated that long-term and high-dose DEX promoted lipid metabolism and chemokine signals, while inhibited glucose metabolism, monocytes recruitment and the differentiation of T lymphocytes.

### RNA-seq analysis of DEGs in the kidney of DEX-injected mice

**Figure 2:**
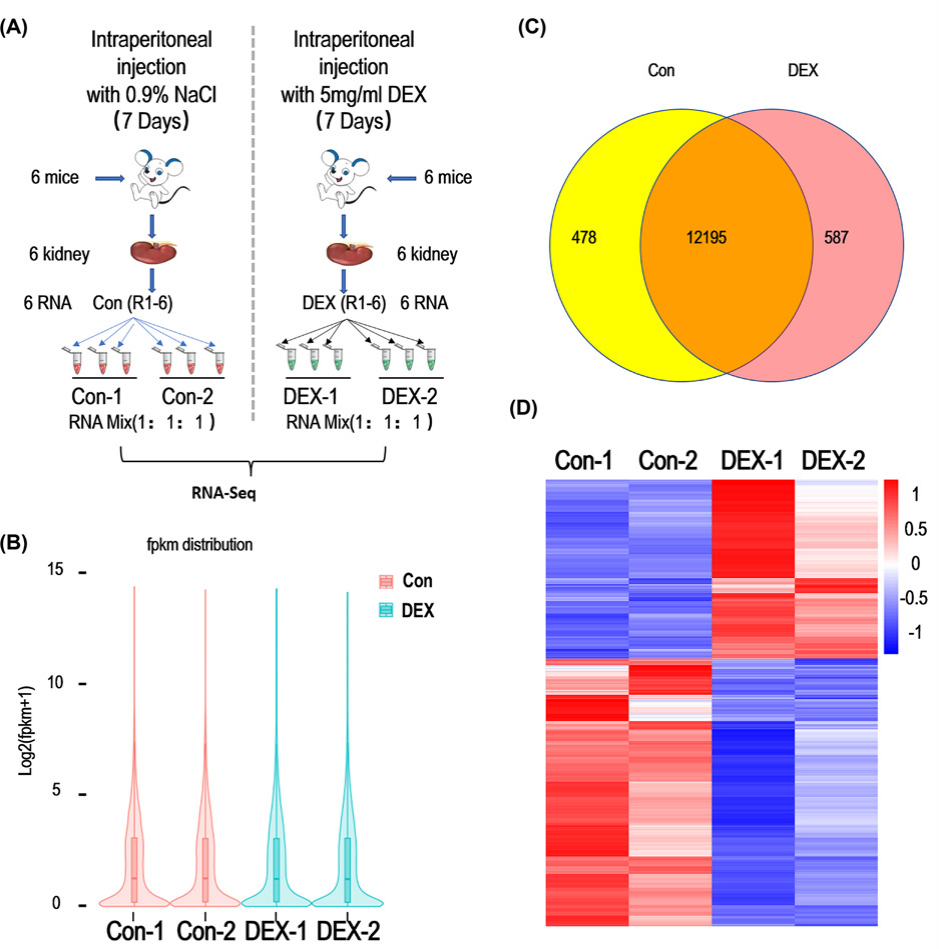
Strategy of RNA-seq analysis and identification of DEGs in mice kidneys with DEX treatment (**A**) Schematic of the kidney RNA-seq analysis strategy (Control: left panel; DEX: right panel; *n*=6, RNA was mixed with 1:1). (**B**) Violin plots showing the expression levels of genes associated with Con-1, Con-2, DEX-1, DEX-2 RNA-seq reads. The y-axis represents the Log_2_ value of fpkm +1. (**C**) Venn diagram shows genes expressed in control and DEX treatment groups. A total of 478 and 587 genes were only detected in control and DEX treatment groups, respectively, and 12195 genes were detected in both groups. (**D**) Heatmap of differentially expressed mRNAs in control and DEX treatment groups. Red represented up-regulated genes while blue represented down-regulated genes.

RNA-seq was used to analyze the transcriptome of mice kidneys from control and DEX treatment groups, which were carried out with two replicates for each group. Overall study design and experimental workflow is shown in Figure 2A. FPKM distribution analysis results showed that there was no obvious difference between the control and DEX treatment groups (Figure 2B). As a result, 12673 and 12782 genes were identified in control and DEX treatment group, respectively, including 12195 genes that were detected in both groups, and 478 and 587 genes were only detected in control and DEX group, respectively. (Figure 2C and Supplementary List S1). For genes expressed in both groups, an adjusted *P*-value <0.05 and log2 (fold change) > 1 were set as the threshold for obtaining significant genes. These results were visualized by a heat map depicting transcripts of DEGs (Figure 2D). The information of RNA-seq and mapping to reference genome were presented in Table 1. Transcriptome data were consistent with our real-time PCR results, which indicated that RNA-seq data were reliable and reproducible.

**Table 1:** Comparative analysis of sequencing reads mapping to the reference genome

Sample name	CON_1	CON_2	DEX_1	DEX_2
**Raw reads**	21931970	22193790	22439925	20262113
**Clean reads**	20647247	20893250	21145128	19596878
**Q20 (%)**	95.64	95.58	95.56	95.71
**Q30 (%)**	89.54	89.37	89.34	89.66
**GC content (%)**	48.05	48.15	48.32	48.21
**Total mapped reads**	19659612	19912166	20213095	18709566
**Uniquely mapped reads**	18001497	18318191	18555762	17206577
**Multiple mapped reads**	1658115	1593975	1657333	1502989
**Total mapping rate**	0.9522	0.9531	0.9559	0.9547
**Uniquely mapping rate**	0.8719	0.8768	0.8775	0.878
**Multiple mapping rate**	0.0803	0.0763	0.0784	0.0767

### DEX influenced biological process of immune system response, lipid metabolism and cell migration

**Figure 3:**
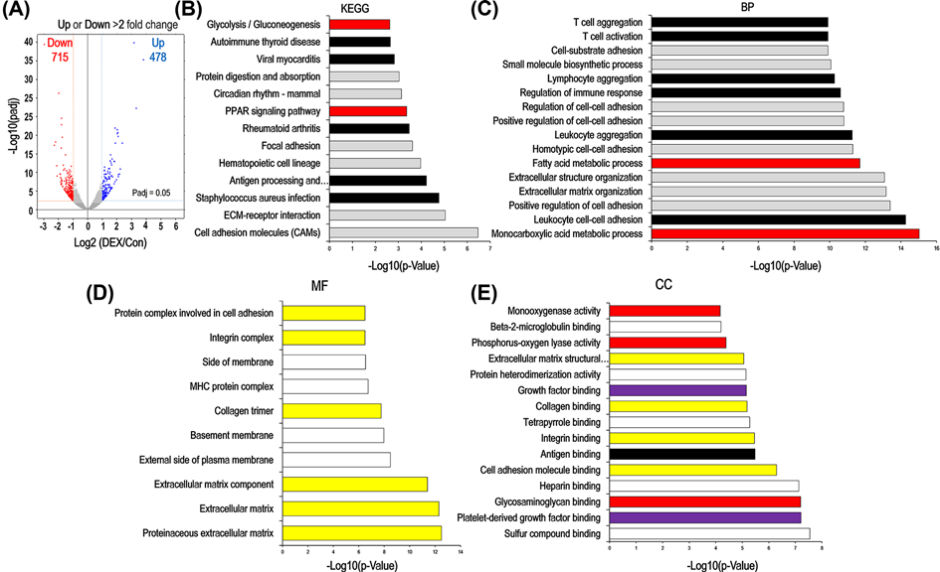
GO and KEGG enrichment of DEGs (**A**) Volcano plots show the distribution of DEGs in control and DEX treatment groups. Red (left) indicates down-regulated gene expression, while blue (right) up-regulated gene expression and black (middle) unchanged. The y-axis represents −Log10 (*P*_adj_), the x-axis represents fold change Log2 (DEX/Con), an adjusted *P*-value of 0.05 and log2 (fold change) of 1 were set as the threshold for significantly differential expression. Differential genes of the RNA-seq compared DEX with Con were analyzed by (**B**) KEGG, (**C**) biological processes (BPs), (**D**) molecular function (MF) and (**E**) cellular component (CC). The x-axis represents −Log10 (*P*-value).

Transcriptomics analysis indicated a total of 1193 DEGs, with 478 up-regulated and 715 down-regulated genes, respectively. A volcano plot was plotted to visualize these DEGs (Figure 3A). To analyze biological impacts of high-dose DEX treatment in kidney, we performed KEGG (shown in Supplementary List S2) and GO (shown in Supplementary List S3) enrichment of DEGs and found 13 KEGG pathways were significantly altered (*P*<0.05; Figure 3B), in which glycolysis and peroxisome proliferator-activated receptor (PPAR) signaling pathways (Figure 3B, red filled columns) were enriched in our profiling, furthermore, immune system response-associated signaling pathways (Figure 3B, black filled columns) were also included. However, cell adhesion molecules and extracellular matrix (ECM)–receptor interaction signaling pathways were most highly enriched in all 13 enriched pathways (Figure 3B). Biological analysis indicated that monocarboxylic acid metabolic and fatty acid metabolic processes (Figure 3C, red filled columns), and immune response-related biological processes were relevant to DEX treatment in mouse kidneys (Figure 3C, black filled columns). Proteinaceous ECM and ECM were the most significant enrichment terms in molecular function category (Figure 3D, yellow fill columns). Sulfur compound binding was the significant enrichment term in cellular components category and cytokine (Figure 3E). Taken together, these results indicate that high-dose DEX treatment affects immune system response, lipid metabolism and cell migration in mice kidneys.

### Cell migration and immune response-related signaling pathways decreased greatly in the kidneys of DEX-injected mice

**Figure 4:**
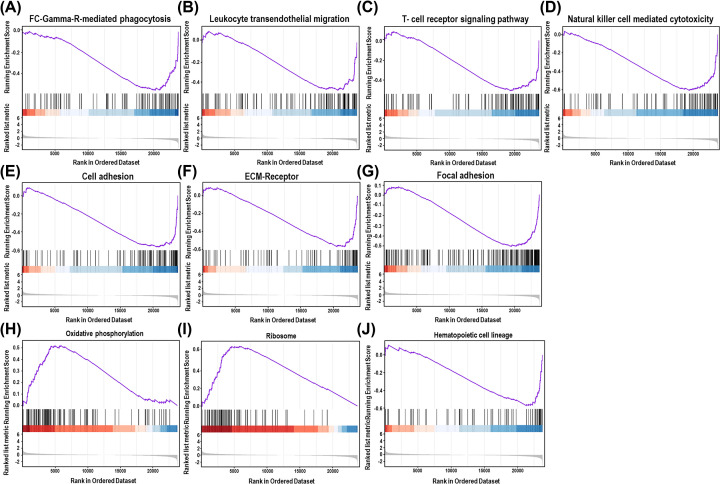
Enrichment plots of indicated gene set obtained by GSEA comparing control and DEX treatment groups (**A–D**) Gene expression profiles were enriched for immune-response gene set, Fc γ R-mediated phagocytosis (A), leukocyte transendothelial migration (B), T-cell receptor signaling pathway (C) and NK cell-mediated cytotoxicity (D) were significantly down-regulated pathways in DEX-treated groups. (**E–G**) Gene expression profiles were enriched for cell adhesion-related gene sets, cell adhesion (E) ECM–receptor interaction (F) and focal adhesion (G) were also down-regulated by DEX treatment. Oxidative phosphorylation (**H**) and ribosome-associated pathways (**I**) were up-regulated and hematopoietic lineage (**J**) was down-regulated in the kidney of DEX-injected mice.

GSEA looking for differentially activated or inhibited pathways in the kidney of DEX-injected mice between saline treatment mice showed that immune response-associated signaling pathways, such as Fc γ R-mediated phagocytosis, leukocyte transendothelial migration, T-cell receptor signaling pathway and NK cell-mediated cytotoxicity were significantly down-regulated pathways in DEX-treated groups (Figure 4A–D), and cell migration-related pathways, such as cell adhesion, ECM–receptor interaction and focal adhesion, were also decreased by DEX treatment (Figure 4E–G). In addition, hematopoietic lineage-associated genes were also decreased in the kidney of DEX-injected mice (Figure 4J). However, in the top ten highly enriched pathways, only oxidative phosphorylation and ribosome-associated pathways were up-regulated in the kidney of DEX-injected mice (Figure 4H,I). All raw data were shown in Supplementary List S4. Collectively, GSEA showed that high-dose DEX treatment suppressed immune response and cell migration, but promoted oxidative phosphorylation response and gene translation in mice kidney.

### DEX treatment dysregulated the expression of multiple protein kinases in mice kidney

**Figure 5:**
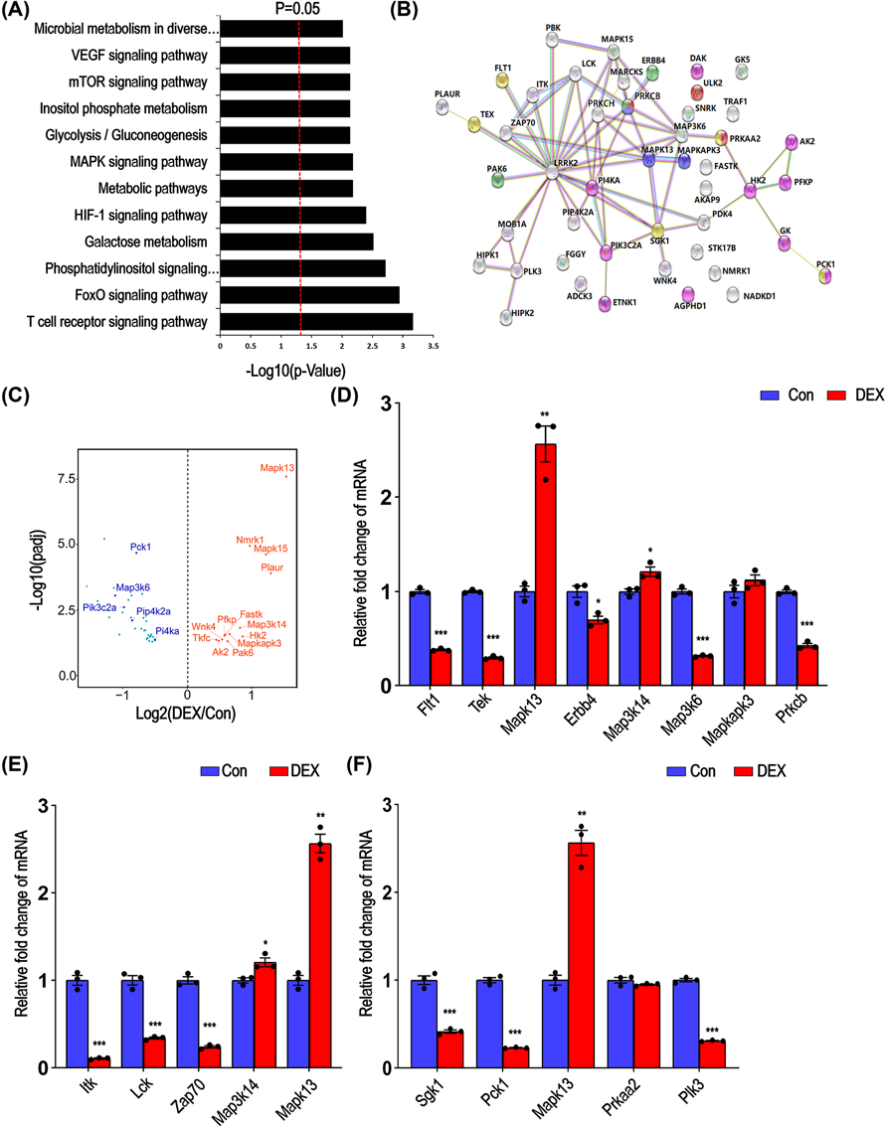
KEGG analysis and validation of DEKs (**A**) KEGG pathway analysis of DEKs, the red dotted line represents *P*=0.05. (**B**) The network of DEK. (**C**) Volcano plots show the distribution of DEKs in control and DEX treatment groups. Red points represent the up-regulated genes and blue represent the down-regulated genes. (**D–F**) The transcriptional levels of kinases related to MAPK signaling pathway, TCR signaling pathway and forkhead box class O (FoxO) signaling pathway were measured by quantitative PCR (blue: control, red: DEX; mean ± s.e.m; *n*=3, *P<0.05, **P<0.01, ***P<0.001, Student’s *t* test).

Previous studies showed that DEX could affect MAPK signaling pathway [30] and NF-κB signaling pathway [31]. Therefore, in order to explore the effect of excessive DEX on related protein kinases in the kidney, we analyzed the expression of protein kinases in the kidney. In DEGs, approx. 47 kinase genes (including 36 serine/threonine protein kinase and 11 tyrosine protein kinases) were changed under DEX treatment (shown in Supplementary List S5). KEGG analysis indicated that these differentially expressed kinases (DEKs) associated with T cell receptor (TCR) signaling pathway: IL2-inducible T cell kinase (Itk), lymphocyte cell-specific protein-tyrosine kinase (Lck), mitogen-activated protein kinase kinase kinase 14 (Map3k14), mitogen-activated protein kinase 13 (Mapk13) and ζ chain of T cell receptor-associated protein kinase 70 (Zap70); forkhead box class O (FoxO) signaling pathway: mitogen-activated protein kinase 13 (Mapk13), phosphoenolpyruvate carboxykinase 1 (Pck1), Polo-like kinase 3 (Plk3), protein kinase AMP-activated catalytic subunit α 2 (Prkaa2), Sgk1; phosphoinositide 3-kinase (PI3K)/AKT signaling pathway: Erb-B2 receptor tyrosine kinase 4 (Erbb4), fms-related receptor tyrosine kinase 1 (Flt1), Pck1, Prkaa2, Sgk1 and endothelial-specific receptor tyrosine kinase (Tek); MAPK signaling pathway: Erbb4, Flt1, Map3k14, mitogen-activated protein kinase kinase kinase (Map3k6), Mapk13, mitogen-activated protein kinase-activated protein kinase 3 (Makpapk3), protein kinase C β (Prkcb) and Tek, and inositol phosphate metabolism signaling pathway: phosphatidylinositol 4-kinase catalytic α polypeptide (Pi4ka), phosphatidylinositol 3-kinase C2 domain containing α polypeptide (Pik3c2a) and phosphatidylinositol 4-kinase type 2 α (Pi4k2a) (Figure 5A and Supplementary List S6). PPI network results showed that multiple kinases can interact with leucine-rich repeat kinase 2 (LRRK2), MAPK13, MAP3K6, protein kinase C η (PRKCH) and SGK1 (Figure 5B). Volcano plot presented the representative significantly up- and down-regulated genes including MAPK and PI3K signaling pathways, suggesting that MAPK signaling pathway and PI3K signaling pathway were affected by high-dose DEX in the kidney of mice (Figure 5C and Supplementary List S7). Quantitative PCR also demonstrated the similar expression pattern of these changed genes in the top three enrichment signaling pathways in the kidney of DEX-injected mice (Figure 5D–F). These results indicated that multiple protein kinases were dysregulated in the kidney of DEX-injected mice.

## Discussion

As an anti-inflammatory, anti-shock and immunosuppressive agent, GCs have a myriad of effects on multiple organ systems. Therefore, the mechanism of long-term and high-dose GC-induced deleterious side effects is complex and has not been fully elucidated.

Here, we constructed a mouse model with high-concentration dexamethasone in serum through intraperitoneal injection of high-dose dexamethasone and analyzed the expression of transcripts in the kidney by RNA-sequence. A total of 10 μg/g body weight/day DEX for 1-week injection disordered glucose and lipid metabolism, inhibited immune response and ECM–receptor interaction in mice kidney without changing the body growth of mice. GSEA enrichment indicated that DEX dominant-negative regulated cell migration and T-cell response, but promoted oxidative phosphorylation and ribosome-associated genes expression in mice kidney. In addition, 48 protein kinases (13 up-regulated and 34 down-regulated) were seriously dysregulated in DEX-treatment mice kidney.

Disorder of sugar and lipid metabolism contributes to multiple side effects of GCs in clinical treatment [32–35]. Intraperitoneal injection of far beyond physiological dose of dexamethasone into mice has obviously affected glycolysis, lipid metabolism, PPAR signaling pathway, cell adhesion and immune-associated pathways in the kidney. These findings are consistent with reported evidence [36–41]. Interestingly, biological process, KEGG and GSEA analyses revealed that far beyond physiological dose of dexamethasone injection negative regulates immune response, cell migration and cell chemotaxis, but promotes oxidative phosphorylation and ribosome-associated genes in the kidney. Evidence suggests that high-dose and long-term injection of dexamethasone decrease the variety and expression of immunoglobulin heavy (light) chain variable region genes in mice spleen [42]. Publications indicate that the increasing of mitochondrial capacity to generate ATP is the main reason for dexamethasone to maintain the efficiency of the mitochondrial oxidative phosphorylation process in liver [43,44]. In patients with Diamond-Blackfan anemia (DBA) and myelodysplastic syndrome (MDS), dexamethasone treatment dysregulates ribosome function and increases the number of erythroid cells produced from normal CD34(+) cells and from CD34(+) cells with the types of ribosome dysfunction [45]. Inhibition of chemotaxis of immune-associated cells explains well for the anti-inflammatory effect of dexamethasone [46,47]. Our findings are highly consistent with these reported publications about dexamethasone function in immune response, oxidative phosphorylation and ribosome function. In dexamethasone-treated mice, 47 kinase genes (36 serine/threonine and 11 tyrosine protein kinases) are significantly ectopic expressed in the kidney. Signaling pathway-enriched analysis indicates that PI3K/AKT and MAPK are the main pathways affected by injection of dexamethasone for 7 days. Activated-PI3K/Akt inhibited-Foxo1 has been demonstrated to decrease the efficiency of G6pc and Pck1-mediated gluconeogenesis in mouse liver [48]. Pdk4 and Pck1 have been down-regulated in goat liver treated with dexamethasone [49]. These reported results are consistent with our findings in the kidney of dexamethasone mice. Interestingly, in all 47 changed protein kinase genes, MAPK13 and MAPK15 are the top fold-changed in up-regulation 13 kinase genes. MAPK13 response to stress and involved in cytokines production, endocytosis, cell migration, chromatin remodeling and transcriptional regulation [50–53], but no publications about its association with dexamethasone. Decrease in MAPK15 in human airway epithelial cells promotes endogenous glucocorticoid receptor expression-stimulated by dexamethasone [54]. As a negative regulator of cell proliferation, dexamethasone induced-MAPK15 expression contributes to the balance control of cell proliferation and apoptosis in kidney. In down-regulated genes, *Lck*, *Plk3*, *Zap70*, *Prkcb*, *Itk* and *Sgk1* are the most decreased protein kinases in the kidney of dexamethasone treated mice. *Lck*, *Zap70* and *Itk* are key regulators of TCR complexes. Their down-regulation explains the inhibition role of dexamethasone in mouse kidney for their regulation on TCR signaling cascade [55–58]. Interestingly, *Sgk1* is widely involved in multiple cellular processes, such as the adjustment of ion channels, the activity of NF-κB and other transcription factors, cell metabolism, inflammation and hormone secretion [59]. However, the vast majority of studies in cells support the conclusion of dexamethasone up-regulation Sgk1 mRNA [60–62]. However, far beyond dose of dexamethasone injection for 7 days decreases the expression of Sgk1 in mice kidney. Exploration of the mechanism underlying this is helpful for researchers to solve the side effects of dexamethasone treatment in clinical therapy.

Although we found that high-dose GCs would affect metabolism and multiple kinase expressions, we did not further explore the mechanism of this situation. Meanwhile, we did not build a specific disease model to study the influence of high-dose GCs on disease, which will be a problem to be solved in the future (Supplementary Figure S2).

## Conclusion

In conclusion, high-dose DEX promoted lipid metabolism and chemokine signals, while inhibited glucose metabolism, monocytes recruitment and the differentiation of T lymphocytes, which was confirmed by qPCR and KEGG and GO enrichment of DEGs. At the same time, 47 kinase genes, including 36 serine/threonine protein kinase and 11 tyrosine protein kinases, were changed under DEX treatment. All of these might further elucidate the mechanism of side effects for high-dose GCs treatment and provide evidence for clinical practice. However, we did not further explore the specific mechanisms by which high-dose GCs lead to changes in glycolipid metabolism, various protein kinases, and different signaling pathways. Meanwhile, we did not build a specific disease model to study the influence of high-dose GCs on disease, which will be a problem to be solved in the future.

## Supplementary Material

Supplementary Figures S1-S2 and Table S1

Supplementary List S1-S7

## Data Availability

We declare that the materials described in the manuscript are available from the corresponding author upon request. All relevant raw and edited data will be freely accessible to any scientist for non-commercial purposes.

## Abbreviations

cDNA: complementary DNA
DEG: differentially expressed gene
DEK: differentially expressed kinase
DEX: dexamethasone
DNA: deoxyribonucleic acid
ECM: extracellular matrix
FPKM: fragments per kilobase of transcript per million mapped fragments
GC: glucocorticoid
GO: Gene Ontology
GSEA: gene set enrichment analysis
H&E: Hematoxylin and Eosin
KEGG: Kyoto Encyclopedia of Genes and Genomes
MAPK: mitogen-activated protein kinase
NF-κB: nuclear factor κβ
NK: natural killer
PI3K: phosphoinositide 3-kinase
PPAR: peroxisome proliferator-activated receptor
PPI: protein–protein interaction
qPCR: real-time quantitative polymerase chain reaction
RNA: ribonucleic acid
RNA-seq: RNA sequencing
TCR: T cell receptor
