# Supplementary material for: High-dose dexamethasone injection disordered metabolism and multiple protein kinases expression in the mouse kidney
Source: Biosci Rep. 2021 Nov 18;41(11):BSR20211847. doi: 10.1042/BSR20211847 (PMC8607334; doi:10.1042/BSR20211847)
Supplement: Supplementary Figures S1-S2 and Table S1 [file BSR-2021-1847_supp.pdf]

Supplementary Figure 1

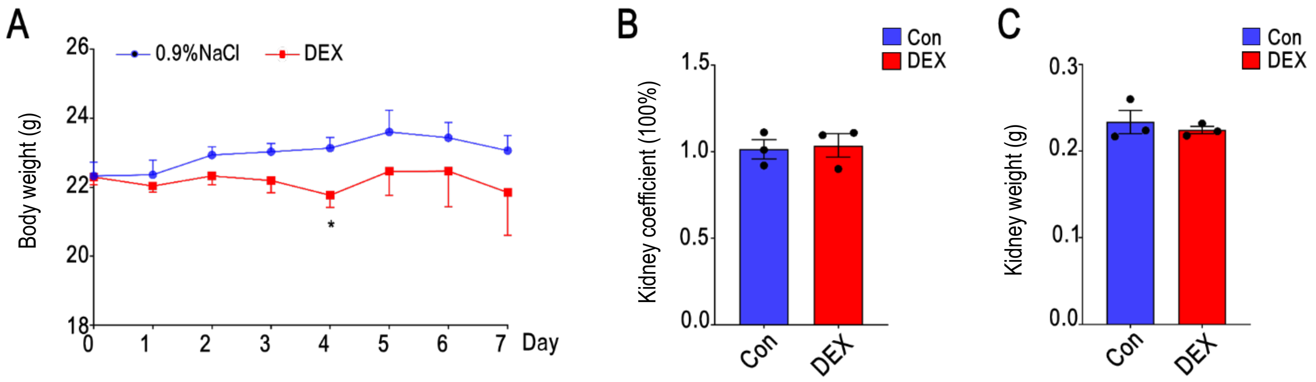

Supplementary Figure 1. Kidney and body weight between control and DEX treatment groups. A, Body weight curves of control (circles) and DEX treatment (squares) groups (Mean ± s.e.m, n = 3 per group, * P <0.05, Student's t-test). B, The kidney coefficient between the kidney weight and body weight. C, The kidney weight between control and DEX treatment group (blue: control, red: DEX; Mean ± s.e.m; n = 3 per group, * P < 0.05, ** P < 0.01, *** P < 0.001, Student's t-test).

Supplementary Figure 2

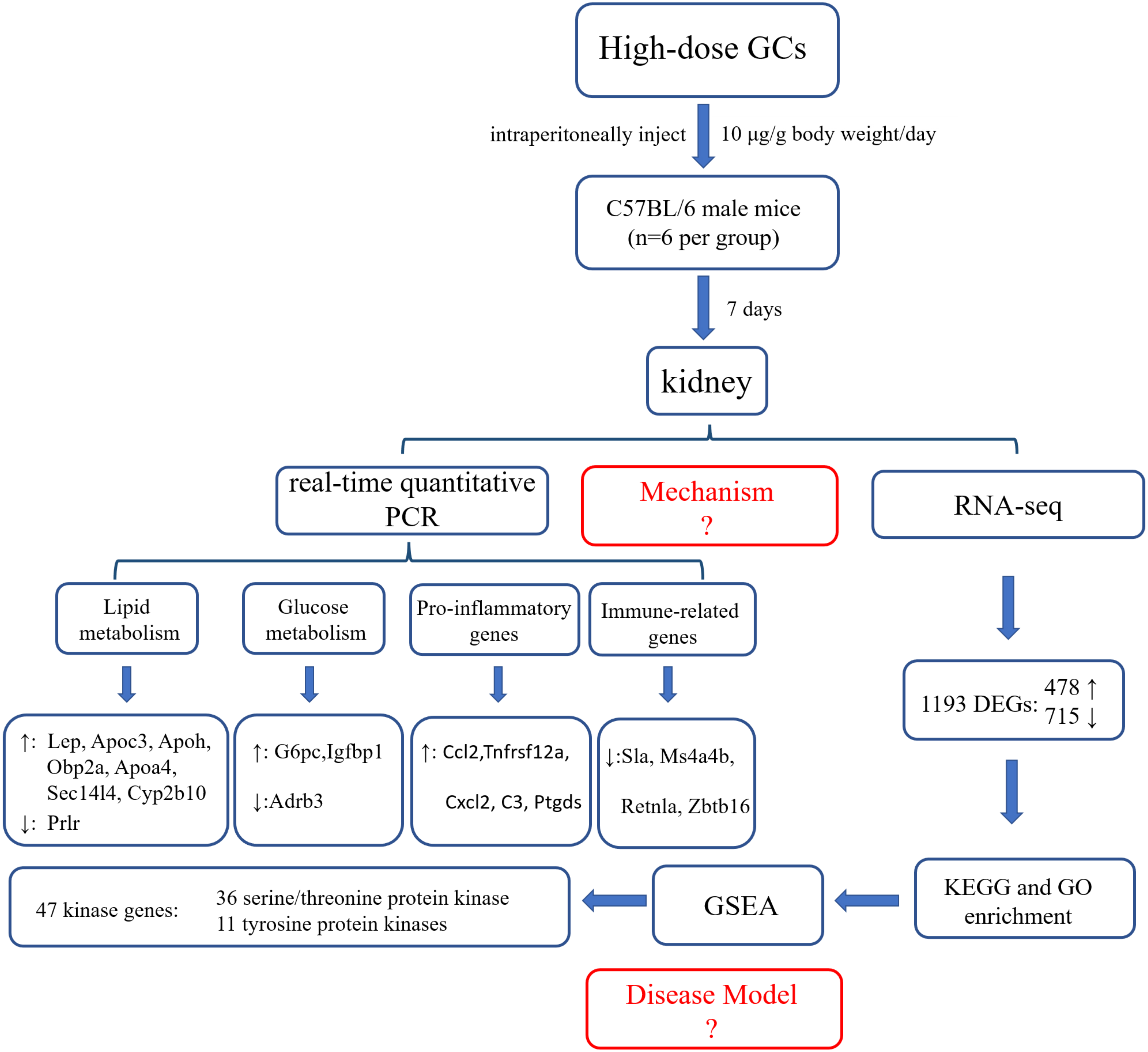

Supplementary Figure 2. A schematic diagram of the study and future directions.

Supplementary Table 1: The quantitative PCR primer sequences used in this study.

Accession number	Gene Name	Primer Name	Sequence (5'---3')
NM_013475.4	Apoh	m.Apoh-F	GCCACACTTTGCCATGATCG
		m.Apoh-R	AGGACCAAGTTCCCGTCTTG
NM_011333.3	Ccl2	m.Ccl2-F	TGCAGGTCCCTGTCATGCTT
		m.Ccl2-R	GGCGTTAACTGCATCTGGCT
NM_001289755.1	Apoc3	m.apoc3-F	GCATCTGCCCGAGCTGAAGA
		m.apoc3-R	AAGCCGGTGAACTTGTCAGTAA
NM_008493.3	Lep	m.Leptin-F	TTCACACACGCAGTCGGTAT
		m.Leptin-R	GATTCTCCAGGTCATTGGCTAT
NM_009999.4	Cyp2b10	m.Cyp2b10-F	CAAAGTCCCGTGGCAACTTCC
		m.Cyp2b10-R	CCCCATTGGCAAAGATCACAC
NM_013462.3	Adrb3	m.Adrb3-F	GTCGTCTTCTGTGTAGCTACGGT
		m.Adrb3-R	TAGCCATCAAACCTGTTGAGC
NM_009140.2	Cxcl2	m.Cxcl2-F	TGCGCTGTCAATGCCTGAAGA
		m.Cxcl2-R	GTTAGCCTTGCCTTTGTTCAGTA
NM_007468.2	Apoa4	m.Apoa4-F	CCAATGTGGTGTGGGATTACTT
		m.Apoa4-R	AGTGACATCCGTCTTCTGAAAC
NM_146013.1	Sec14l4	m.Sec14l4-F	ATGAGAGGACAAGTCGGGGAC
		m.Sec14l4-R	CCATCGCAGAAGGAAGTAGTCA
NM_009778.3	C3	m.C3-F	CCAGCTCCCCATTAGCTCTG
		m.C3-R	GCACTTGCCTCTTTAGGAAGTC
NM_013749.2	Tnfrsf12a	m.Tnfrsf12a-F	GTGTTGGGATTCGGCTTGGT
		m.Tnfrsf12a-R	GTCCATGCACTTGTCGAGGTC
NM_011169.5	Prlr	m.Prlr-F	GGTTGCCACCTACCATAACTGA
		m.Prlr-R	AGTATCCATGGTCTGGCTTGCA
NM_008341.4	Igfbp1	m.Igfbp1-F	ATCAGCCCATCCTGTGGAAC
		m.Igfbp1-R	TGCAGCTAATCTCTCTAGCACTT
NM_001029841.4	Sla	m.Sla-F	ATGGGGAATAGCATGAAATCCAC
		m.Sla-R	GGAGATGGGTAGTCAGTCAGC
NM_030712.4	Cxcr6	m.Cxcr6-F	GAGTCAGCTCTGTACGATGGG
		m.Cxcr6-R	CCTTGAACTTTAGGAAGCGTTTG
NM_008061.4	G6pc	m.G6pc-F	CGACTCGCTATCTCCAAGTGA
		m.G6pc-R	GTTGAACCAGTCTCCGACCA
NM_021718.2	Ms4a4b	m.Ms4a4b-F	TTCTGCCTTGCTGTGTCCGTCT
		m.Ms4a4b-R	ACACATTTCCTGGAACATTGGTC
NM_009349.3	Inmt	m.Inmt-F	GCAGAGCAGGAAATCGTAAAGT
		m.Inmt-R	GGGGTGTAGTCAGTGACAATGAT
NM_011361.3	Sgk1	m.Sgk1-F	TGGCACGCCTGAGTATCTGG
		m.Sgk1-R	GTCGTACATCTCAGCCGTGTT
NM_008454.3	Klk1b16	m.Klk1b16-F	CCACTGCTATGTCGATGAGTGT
		m.Klk1b16-R	GCCCCAGGAGGTAACTTTTCAA
NM_008963.3	Ptgds	m.Ptgds-F	GCTCTTCGCATGCTGTGGAT
		m.Ptgds-R	GCCCCAGGAACTTGTCTTGTT
NM_153558.1	Obp2a	m.Obp2a-F	GACCTGGTGGATTACTCTGGG

		m.Obp2a-R	CAACTGTGGTCTCCAAGTCTCC
NM_007939.2	Eph receptor A8	m.Epha8-F	AGACCTGCCGTTGTGACCTCA
		m.Epha8-R	CACACTGCGTTGTAGGTGATGT
NM_020509.4	Retnla	m.Retnla-F	CTTCTTGCCAATCCAGCTAACTA
		m.Retnla-R	GCAGTGGTCCAGTCAACGA
NM_001033324.3	Zbtb16	m.Zbtb16-F	GGTTCCTGGACAGTTTGCGAC
		m.Zbtb16-R	TGAGAACTGGGCACCGCATT
NM_011065.5	Per1	m.Per1-F	TCTCATAGTTCCTCTTCTGGCA
		m.Per1-R	CTGTGAGTTTGTACTCTTGCTG
NM_010228.4	Flt1	m.Flt1-F	GCTCTACACCTGTCGCGTGAA
		m.Flt1-R	ATGGACAGCCGATAGGACCGT
NM_001290549.1	TEK	m.Tek-F	GAGAACAACATAGGATCAAGCA
		m.Tek-R	ATAATCAGAAACGCCAACAGCA
NM_011950.2	MAPK13	m.Mapk13-F	GCTCAAAGGTCTAAAGTACATCC
		m.Mapk13-R	CACCACATAGCCCGTCATCT
NM_010154.2	Erbb4	m.Erbb4-F	CCACAGAAAATCACTGCCAGAC
		m.Erbb4-R	AGTAACGCAGGCTCCACTGTC
NM_016896.3	Map3k14	m.Map3K14-F	GGCGAGGTCCACAGAATGAAGG
		m.Map3K14-R	GCCTTCTCTCACAGCTCCATAG
NM_178907.3	Mapkapk3	m.Mapkapk3-F	CGCATCCTGGACGTGTATGAG
		m.Mapkapk3-R	CAATGTCCCGCATTATCTCTGCA
NM_001281967.1	Itk	m.Itk-F	TCTCCTGTATGTGTTTGCTCCA
		m.Itk-R	GCATTCTTGGATGGGTCGTAG
NM_001162433.1	Lck	m.Lck-F	TGGAGAACATTGACGTGTGTG
		m.Lck-R	GCGATAACCAGGTTGTCTTGCA
NM_001289612.1	Zap70	m.Zap70-F	GGCCAGAAGCCCTACAAGAA
		m.Zap70-R	GTTCCGCATACGTTGTTCCACA
NM_011044.3	Pck1	m.Pck1-F	CATCTTCACCAACGTGGCCGA
		m.Pck1-R	GCACATGGTTCCGCGTCCTG
NM_178143.2	Prkaa2	m.Prkaa2-F	CTGAAGCCAGAGAATGTGCTGC
		m.Prkaa2-R	GAGATGACCTCAGGTGCTGCAT
